# First known case of catatonia due to cyclosporine A-related neurotoxicity in a pediatric patient with steroid-resistant nephrotic syndrome

**DOI:** 10.1186/s12888-019-2107-6

**Published:** 2019-04-24

**Authors:** R. David Heekin, Kalonda Bradshaw, Chadi A. Calarge

**Affiliations:** 1 Department of Psychiatry and Behavioral Sciences, Baylor College of Medicine, Houston, TX USA; 2 University of Texas Health Science Center at Houston, Houston, TX USA

**Keywords:** Catatonia, Nephrotic syndrome, Cyclosporine, Neurotoxicity, Propofol

## Abstract

**Background:**

Catatonia is a neuropsychiatric syndrome characterized by diverse psychomotor abnormalities, including motor dysregulation and behavioral and affective disturbances. Once thought to occur primarily in the context of schizophrenia, recent data suggest most cases of catatonia develop in individuals with depressive or bipolar disorders. Moreover, catatonia may ensue in general medical and neurological conditions, as well as due to a variety of pharmaceuticals, drugs of abuse, and toxic agents. At one time considered rare in pediatric patients, evidence now suggests catatonia is both underrecognized and undertreated in this population, where it carries an elevated risk of morbidity and mortality. Here we present the case of a child with steroid-resistant nephrotic syndrome who developed catatonia due to cyclosporine A-related neurotoxicity.

**Case presentation:**

A 9-year-old African-American boy with no psychiatric history and a 9-month history of nephrotic syndrome due to focal segmental glomerulosclerosis was admitted to the local children’s hospital for management of mutism, posturing, insomnia, gait abnormalities, and somatic delusions. Seven days prior to admission, his cyclosporine plasma concentration was elevated at 1224 ng/mL (therapeutic range: 100–200 ng/mL). Upon admission, cyclosporine was discontinued and psychiatry was consulted, diagnosing catatonia. The patient subsequently received propofol 80 mg IV resulting in a transient lysis of catatonia. Over a lengthy hospitalization, the patient’s catatonia was initially treated with lorazepam, quetiapine being added later to target psychosis. All signs and symptoms of catatonia resolved, and the patient was eventually tapered off both lorazepam and quetiapine with no return of symptoms more than 6 months later.

**Conclusions:**

To our knowledge, this case represents the first reported instance of cyclosporine A-induced catatonia in a patient with steroid-resistant nephrotic syndrome. It illustrates the importance of maintaining vigilance for signs and symptoms of cyclosporine A-related neurotoxicity (including catatonia) in patients with steroid-resistant nephrotic syndrome. In addition, it highlights the challenges faced by clinicians in jurisdictions that prohibit the use of electroconvulsive therapy in pediatric patients.

## Background

Catatonia is a neuropsychiatric syndrome characterized by diverse psychomotor abnormalities, including motor dysregulation and behavioral and affective disturbances. For much of the twentieth century, catatonia was believed to occur primarily in the context of schizophrenia. However, recent data suggest catatonia develops more commonly in individuals with depressive or bipolar disorders [1]. Moreover, it is now well established that catatonia may ensue in the context of general medical and neurological conditions, as well as due to a variety of pharmaceuticals, drugs of abuse, and toxic agents [2–4].

Once thought to be rare in pediatric patients, evidence now suggests catatonia is both underrecognized and undertreated in this age group [5]. In fact, catatonia is more likely to be associated with developmental disorders and general medical conditions in children compared to adults [5]. Significantly, catatonia carries an elevated risk of morbidity and mortality, partly attributed to failure of timely recognition and initiation of appropriate treatment [6]. Here we present the case of a child with steroid-resistant nephrotic syndrome (SRNS) who developed catatonia likely secondary to cyclosporine A (CsA)-related neurotoxicity.

## Case presentation

The patient is a 9-year-old obese African-American boy with a 9-month history of nephrotic syndrome due to focal segmental glomerulosclerosis (FSGS), diagnosed by renal biopsy. He had no prior psychiatric history, although two maternal great aunts had been diagnosed with schizophrenia. His home medications included CsA 125 mg QAM and 100 mg QHS, prednisolone 60 mg daily, amlodipine 5 mg BID, and lisinopril 5 mg daily. He was admitted to the local children’s hospital for management of prolonged staring, mutism, unusual arm posturing, insomnia, and abnormal gait. Significantly, 7 days prior to presenting to our facility, his CsA plasma concentration was 1224 ng/mL (therapeutic range: 100–200 ng/mL) on routine testing. Around this time, according to his parents, the patient had become increasingly anxious. They noted he repeatedly expressed concerns about having “a hole in my neck” and that, when he swallowed, it felt “like it’s only going down one side.” His parents brought him to an outside ED where a rapid strep test was positive and a computed tomography (CT) scan of the head was unremarkable. He was treated with penicillin G and discharged home.

Upon presentation to our facility, the child was alert and grossly oriented. Physical exam was significant for signs of generalized volume overload, with 2+ lower extremity pitting edema. He displayed significant negativism, keeping his eyes closed when the examiner attempted to open them, refusing to open his mouth to command or prying, and biting down hard on a tongue depressor. Vital signs were within normal limits. Initial laboratory exam including blood chemistry, CBC, blood culture, HSV PCR, urine toxicology, and thyroid function tests was remarkable only for hypoalbuminemia. Plasma CsA level was 64 ng/mL. Serum autoimmune encephalitis panel was obtained, returning negative one week later. Chest x-ray was unremarkable. Head CT scan and a magnetic resonance imaging (MRI) scan of the brain with and without contrast were remarkable only for mild-to-moderate prominence of cerebrospinal fluid (CSF) spaces and mild thinning of the corpus callosum without focal abnormalities. At that time, the patient’s home CsA was discontinued due to concern for potential neurotoxicity, and intravenous (IV) methylprednisolone 48 mg daily was substituted for oral (PO) prednisolone as the patient refused to swallow, repeatedly spitting out his medications. He was started on empiric acyclovir, vancomycin, and ceftriaxone for possible meningitis. The initial differential diagnosis was broad and included toxic/iatrogenic causes of psychosis and altered mental status (especially corticosteroids and CsA), primary psychiatric disorders, infectious and autoimmune causes of encephalitis, seizures, vitamin deficiency (e.g. B6, B12, or thiamine deficiency, which may be associated with nephrotic syndrome), or malignancy.

Over the first 24 h of admission, the patient displayed further mental status changes, sleep disturbance, and episodic agitation and confusion. He appeared to be responding to internal stimuli, sitting up in bed suddenly, crying, kicking, and punching indiscriminately. During these times, he would not follow commands, while at times displaying echolalia and verbigeration. He became progressively more agitated, pulling out his IV line, biting off his identification bracelet, and yelling profanity at his mother. This behavior was in stark contrast to the patient’s usually shy and reserved demeanor. During the next 24 h, he exhibited Cotard delusion, telling his parents he had died from “a bomb in my neck” and that he was now a “zombie.” Throughout that day, he displayed increasing mutism and odd mannerisms, raising his hands above his head and out to his sides as if meditating. Psychiatry was consulted and diagnosed catatonia, recommending treatment with lorazepam. However, the patient’s primary team elected to defer this treatment.

On hospital day 3, the patient received propofol 80 mg IV in preparation for a lumbar puncture for further work-up. Within seconds, he became alert and sat up in bed saying, “I feel better now.” He followed commands and was oriented to person, age, place, and year. He recognized his nephrologist by name and was able to recall discussions between his medical team and his parents from earlier in the admission. This drastic change in mental status lasted only a few minutes but led the team to abort the lumbar puncture, ascribing a “behavioral” cause to his presentation and deeming an infectious or autoimmune process unlikely. Factors thought to support this contention included absence of fever, normal laboratory tests, and a months-long corticosteroid treatment (felt to be protective against an autoimmune process).

Our psychiatry consult/liaison team was brought back on board and performed a lorazepam challenge test with 2 mg IV, resulting in temporary resolution of catatonic symptoms and markedly improved mental status. Lorazepam was continued at 2 mg IV 3 times/day (TID). Catatonic symptoms returned on hospital day 4, with the patient displaying alternating mutism, excitement, immobility, stereotypic movements, echolalia, verbigeration, odd mannerisms, negativism, and mild rigidity. His Bush-Francis Catatonia Rating Scale (BFCRS) score was 26. At this point he began to show autonomic instability with a temperature of 37.9 °C, systolic blood pressure ranging from 95 to 147 mmHg over a period of 1 h, and heart rate fluctuating between approximately 60 and 160 bpm over 2 to 3 min despite lying supine and motionless. Lorazepam was increased to 3 mg IV TID with resolution of autonomic instability. On hospital day 5, with close consultation between the primary general pediatrics team, nephrology, and psychiatry, mycophenolate mofetil 500 mg IV BID was started as an alternative to the patient’s home CsA for SRNS, given CsA’s known neurotoxicity risk [7]. Mycophenolate mofetil was subsequently switched to oral (PO) formulation and titrated to 1000 mg PO BID over the following 10 days.

By hospital day 7, the BFCRS score was 11. Over the following 3 weeks lorazepam, was switched to PO formulation and increased to a maximum dose of 3.75 mg PO TID. Despite the increase, the patient continued to show only partial resolution of catatonic symptoms, with continued negativism, slowed movements, somatic delusions, and periodic visual illusions (mistaking wrinkles in his bedsheets for snakes). Due to psychomotor slowing and sedation, lorazepam was decreased to 3 mg PO TID. Due to concern about corticosteroids also potentially causing catatonia and psychosis [8], methylprednisolone was gradually decreased from 48 mg daily to 20 mg daily, then switched to prednisone 25 mg PO daily on day 20. Additionally, mycophenolate mofetil was held for 5 days between days 20–24 as the patient’s parents reported the patient’s mental status seemed to deteriorate when he was given this medication; however this hiatus did not result in any clinical improvement.

After several family and multidisciplinary treatment team meetings, prompted by parents’ concerns about adverse drug effects and potential for inducing neuroleptic malignant syndrome (NMS) in catatonic patients, quetiapine 12.5 mg QHS was started as an adjunctive treatment for catatonia and psychosis on hospital day 28. Over the following 3 weeks, quetiapine was switched to extended-release formulation and titrated up to 300 mg at bedtime, with lorazepam continued at 3 mg PO TID. The patient demonstrated gradual improvement in catatonic signs/symptoms, and by hospital day 39 he was increasingly interactive (although with continued paucity of speech and increased speech latency), no longer exhibiting somatic delusions or sensory misperceptions. By hospital day 68 all signs/symptoms of catatonia had resolved, and the patient’s parents felt he was at his mental status baseline. He was discharged home on day 78, having remained as an inpatient to manage volume overload due to nephrotic syndrome. Psychotropic medications at discharge were lorazepam 3 mg TID and quetiapine extended-release 250 mg QHS. Over the following 3 months the patient was tapered off both medications, with no recurrence of symptoms six months later (Table 1).

**Table 1:** Summary of clinical course

Hospital day	Clinical summary/therapeutic interventions
1	Patient admitted due to staring, mutism, posturing, insomnia, gait abnormalities, and somatic delusions. Home CsA discontinued due to concern for neurotoxicity. Methylprednisolone 48 mg IV daily substituted for home prednisolone 60 mg PO daily. Empiric acyclovir, vancomycin, and ceftriaxone given for possible meningitis.
3	Temporary lysis of catatonia achieved with propofol 80 mg IV. Lorazepam challenge test (2 mg IV one-time dose) later resulted in temporary resolution of catatonic symptoms. Lorazepam 2 mg IV TID started.
4	Due to concern for malignant catatonia, lorazepam increased to 3 mg IV TID with resolution of autonomic instability.
5	Mycophenolate mofetil 500 mg IV BID started as alternative to home CsA for steroid-resistant nephrotic syndrome.
10	Lorazepam 3 mg IV TID switched to 3 mg PO TID.
12	Mycophenolate mofetil 500 mg IV BID switched to 750 mg PO BID.
14	Methylprednisolone decreased to 30 mg IV daily.
15	Mycophenolate mofetil increased to 1000 mg PO BID.
18	Lorazepam increased to 3.75 mg PO TID. Methylprednisolone decreased to 20 mg IV daily.
20	Methylprednisolone 20 mg IV daily switched to prednisone 25 mg PO daily. Mycophenolate mofetil held due to continued altered mental status.
24	Lorazepam decreased to 3 mg PO TID due to concern for psychomotor slowing and sedation.
25	Mycophenolate mofetil 1000 mg PO BID restarted due to no improvement in mental status while off this medication.
28	Quetiapine 12.5 mg PO QHS started as adjunctive treatment for catatonia and psychosis. Quetiapine titrated to 25 mg QAM + 50 mg QHS over the following 7 days.
36	Quetiapine switched to quetiapine XR 100 mg PO QHS. Quetiapine XR titrated to 300 mg PO QHS over the following 12 days.
39	Patient was increasingly interactive and no longer exhibited somatic delusions or sensory misperceptions. Psychotropic meds were lorazepam 3 mg PO TID and quetiapine XR 150 mg PO QHS.
68	Catatonic symptoms noted to have resolved. Parents believed patient was at his mental status baseline. Psychotropic meds were lorazepam 3 mg PO TID and quetiapine XR 300 mg QHS.
78	Patient was discharged home on psychotropic meds lorazepam 3 mg PO TID and quetiapine XR 250 mg PO QHS (both tapered and discontinued over the following 3 months).

*BID*: 2 times per day, *CsA*: cyclosporine A, *IV*: intravenous, *PO*: oral, *QAM*: every morning, *QHS*: every night, *TID*: 3 times per day, *XR*: extended release

## Discussion

We report the first known case of catatonia due to CsA-related neurotoxicity in a patient with SRNS, though we recognize other patients with similar symptoms may have been diagnosed with delirium or psychosis. CsA is the most frequently used immunosuppressive agent for the treatment of SRNS in childhood [9]. CsA-related central neurotoxicity has been extensively documented in both adult and pediatric bone marrow and solid organ transplant recipients (with an incidence of 0.5 to 35%) [10]. In SRNS, however, CsA-related neurotoxicity has been less often described, with posterior reversible encephalopathy syndrome (PRES) appearing to be the most common presentation [11, 12]. In contrast, only a handful of cases of non-PRES CsA-related neurotoxicity in patients with SRNS have been reported. The neurologic signs and symptoms in these cases have included headache, anxiety, tremor, confusion, seizures, coma, and hallucinations (Table 2).

**Table 2:** Reported cases of CsA-related neurotoxicity (excluding PRES) in patients with SRNS

Reference	Age (years)/ Gender	Renal diagnosis	CsA plasma level at time of episode (ng/mL)	Other medications at time of episode	Neurologic signs/symptoms	EEG findings	CT/MRI findings
[9]	4/F	SRNS MesPGN	132	Not reported	Altered conciousness, seizures, cortical blindness	n/a	MRI: Increased T2 intensity and restricted diffusion on DWI/ADC in P, O, W, G
[9]	11/M	SRNS FSGS	141	Not reported	Altered conciousness, seizures	n/a	MRI: Increased T2 intensity and restricted diffusion on DWI/ADC in P, Fr, W
[9]	15/F	SRNS IgA nephropathy	263	Not reported	Altered conciousness, seizures	n/a	CT: hypodense zones in P, O
[10]	6.5/F	SRNS MCD	172	Prednisolone (35 mg/day) Sodium valproate (1 mg/kg IV per hour) Phenobarbital (10 mg/kg IV)	Headache, confusion, hallucinations, seizures, coma	Focus of slow paroxysmal activity in posterior right leads	CT: normal MRI: contrast enhancement in corticosubcortical areas of P and Fr bilaterally (resolved at 3 month comparison study)
[10]	12/F	SRNS MesPGN	203	Prednisolone (60 mg every other day)	Headache, confusion, seizures, status epilepticus	Slow activity (consistent with therapeutic coma) with left occipital spikes	CT: normal MRI: Increased T2 intensity in cortex and cortico-subcortical junction of medial left P and bilateral Fr
[13]	10/M	SRNS FSGS	85.55	Prednisone (2 mg/mg/day)	Anxiety, tremor, headache, neck pain	Normal	CT: “small widening sulcus on brain surface”

*CsA:* cyclosporine A, *PRES:* posterior reversible encephalopathy syndrome, *SRNS:* steroid-resistant nephrotic syndrome, *EEG:* electroencephalogram, *CT:* comptuted tomography, *MRI:* magnetic resonance imaging, *MesPGN:* mesangial proliferative glomerulonephritis, *T2:* T2-weighted imaging, *DWI:* diffusion-weighted imaging, *ADC:* apparent diffusion coefficient mapping, *P:* parietal region, *O:* occipital region, *W:* white matter, *G:* gray matter, *FSGS:* focal segmental glomerulosclerosis, *Fr:* frontal region, *MCD:* minimal change disease, *IV:* intravenous

The onset of neurotoxic symptoms ranges from 1 day to 3 years following the initiation of CsA; however, most adverse effects are noted in the first month and one-third within the first week [14]. Risk factors include hepatic or renal dysfunction, hypomagnesemia, hypocholesterolemia, high-dose glucocorticoid therapy, arterial hypertension, cancer chemotherapy, cerebral ischemia or hemorrhage, and opportunistic infection [14]. Neuroimaging may reveal cerebral white matter hypodensity on CT, corresponding to areas of increased signal on MRI T2 sequence [10].

The exact pathogenesis of CsA neurotoxicity is unclear, with proposed mechanisms including damage to endothelial cells leading to release of vasoactive agents, resulting in vasospasm and microvascular damage [9]. Direct toxicity of CsA has also been proposed; however neurotoxic symptoms do not appear to be dose-dependent, potentially occurring even when CsA plasma concentrations are within the therapeutic range [9]. Among organ-transplant recipients, a wide range of neurological signs and symptoms have been reported. Minor side effects include headache, anxiety, tremor, and insomnia. More serious findings include confusion, delirium, aphasia, dystonia, hallucinations, visual disturbances, akinetic mutism, parkinsonism, peripheral neuropathies, seizures, coma, intracranial hemorrhage, PRES, and cortical blindness [7].

As for catatonia, this has been reported previously in a 54-year-old female bone marrow transplant recipient on CsA [15]. Additionally, in a case series describing three liver transplant patients treated with CsA, multiple signs consistent with catatonia (e.g. immobility, mutism, rigidity, staring, grimacing, and posturing) were reported but attributed to akinetic mutism [16]. Another case report described a patient taking CsA for resistant atopic dermatitis who developed features consistent with catatonia but ultimately was diagnosed with irreversible abulia [17]. To our knowledge, however, catatonia has never been reported in a patient receiving CsA for SRNS, like our patient. Of course, it is possible other causes may have contributed to the development of his catatonia, chief among them being corticosteroids. However, the patient had been maintained on a stable dose of corticosteroids for months prior to developing catatonic symptoms, and he was continued on steroid treatment throughout his care. Nevertheless, given that corticosteroid-induced psychiatric symptoms can emerge at any time during treatment, this cannot be ruled out as a contributing factor in our patient’s presentation [8]. Autoimmune encephalitis, including anti-N-methyl-D-aspartate (NMDA) receptor encephalitis, is another possibility, given the relatively high incidence of catatonia in this condition [18, 19]. While our patient did test negative for autoimmune/paraneoplastic autoantibodies on serum evaluation, CSF testing for anti-NMDA receptor IgG autoantibodies (which our patient did not undergo) is both more sensitive and specific than serum testing [20]. Nevertheless, serum immunohistochemistry-testing for anti-NMDA receptor antibodies is highly sensitive [21]. Arguing in favor of CsA treatment as the likely cause of catatonia in our patient is the fact that he manifested prodromal symptoms of CsA-related neurotoxicity (i.e. heightened anxiety) in close temporal proximity to having elevated CsA plasma concentration on routine testing.

Catatonia may develop due to treatment with a variety of pharmaceutical agents, including fluoroquinolones, cephalosporins, dopamine antagonists (e.g. antiemetics and antipsychotics), corticosteroids, disulfiram, baclofen, tacrolimus, and cyclosporine [5]. In addition, catatonia has been observed in patients withdrawing from GABA-ergic drugs such as benzodiazepines, zolpidem, and gabapentin, as well as with discontinuation of dopamine agonists including bromocriptine and levodopa/carbidopa [5]. Such medication-related presentations, together with catatonia due to general medical and neurological conditions or toxic agents (e.g. carbon monoxide poisoning), are classified under the ICD-10 diagnosis “organic catatonic disorder” (F06.1), which excludes presentations due to alcohol or drugs of abuse [22]. As DSM-5 does not contain a “substance/medication-induced” diagnostic classification for catatonia, our patient’s case was diagnosed as the less-descriptive “other medication-induced movement disorder” [23]. Treatment of “organic catatonia” under its various names involves removing suspected offending agents and/or treating the underlying medical condition and/or drug withdrawal. Otherwise, treatment follows the same approach as for catatonia due to psychiatric illness, including use of benzodiazepines, augmentation with agents such as memantine or amantadine, and electroconvulsive therapy (ECT) [5].

Lysis of catatonia by propofol has previously been reported in a 58-year-old bone-marrow transplant recipient, with catatonia likely precipitated by adenovirus limbic encephalitis in the context of benzodiazepine withdrawal [24]. Propofol (2,6-diisopropylphenol) is a short-acting IV anesthetic approved by the United States Food and Drug Administration (FDA) for the maintenance of anesthesia in children ≥2 months of age and for induction of anesthesia in children ≥3 years of age [25]. It is also frequently used off-label by anesthesiologists and critical care physicians for sedation for procedures and imaging in pediatric patients [26]. Propofol’s mechanism of action involves positive modulation of the inhibitory function of γ-aminobutyric acid (GABA) through binding at GABA_A_ receptors [27]. In addition, propofol inhibits the N-methyl-d-aspartate (NMDA) glutamate receptor and reduces calcium influx through slow calcium-ion channels [28]. Given its rapid onset and brief duration of action, future research should investigate propofol’s utility as an alternative challenge for catatonia. The use of ECT in combination with propofol anesthesia as a means of increasing efficacy in catatonic patients should also be examined.

Several historical circumstances have combined to obscure the recognition of catatonia as a discrete pathological entity, including most notably its incorporation by Emil Kraepelin into the concept of *dementia praecox* in the 1890s (later termed “schizophrenia” by Eugen Bleuler) as well as the later deinstitutionalization shift within the field [19, 29]. Given its mercurial status as a diagnostic entity, it is hardly surprising that many patients with catatonia go all too often unrecognized by clinicians [30]. This challenge manifests even more acutely in pediatric cases, where catatonic symptoms may be misdiagnosed as selective mutism, autism spectrum disorder, somatic symptom disorder, or malingering once schizophrenia has been ruled out. A retrospective chart review of 101 inpatients at an academic pediatric psychiatric facility found evidence of 3 or more catatonic signs in 18 patients (17%), while treating clinicians diagnosed catatonia in only two cases [31]. In the case of our patient being treated at a large academic tertiary referral hospital, the question put to the psychiatry consult service was whether his observed abnormalities (e.g. mutism, negativism, echolalia, stereotypies) might be “behavioral” in nature, after his dramatic and paradoxical response to propofol infusion. This likely reflects the poorly understood nature of catatonia among clinicians. This situation might be remedied by more collaborative efforts in medical education to promote knowledge of catatonia and the importance of early specific treatment, as delays in treatment are associated with increased morbidity and mortality—a circumstance even more unfortunate as the initial recommended treatment, lorazepam, carries minimal risk [4]. As consult-liaison psychiatrists establish a more widespread presence in hospital settings, they could play a key role in promoting increased awareness of catatonia across medical specialties.

Our patient achieved only partial remission with lorazepam, whose dose could not be increased further due to sedation. Quetiapine subsequently was used as an adjunctive treatment to target both catatonia and psychosis. While both first and second-generation antipsychotics (including clozapine) have been associated with development of catatonia and malignant catatonia/NMS, second-generation antipsychotics also have been used to treat catatonia [5]. Several case reports have documented successful treatment of catatonia with olanzapine, either as the primary agent or combined with more standard treatments such as ECT, benzodiazepines, or amantadine [5, 32–42]. This has led to olanzapine being recommended as a preferred antipsychotic to use in catatonia, if necessary [5]. After discussion of risks and benefits we chose quetiapine, which has been used successfully to treat catatonia, in part due to its less problematic cardiometabolic risk profile for our obese patient [43]. We also speculated quetiapine might be less likely to induce NMS/malignant catatonia than antipsychotics with stronger dopamine D_2_ receptor antagonist properties.

Finally, when evidence of autonomic instability emerged, the use of ECT in a prepubescent child with potential malignant catatonia was considered. In the state of Texas, however, the use of ECT in children under 16 years of age is prohibited, even in life-threatening circumstances (similar restrictions exist in California and Colorado) [44]. Such laws have the potential to deprive patients and their families of established life-saving treatments, as in malignant catatonia, which carries a 10–20% mortality rate when untreated [45]. Moreover, without recourse to ECT, which may produce more rapid response and remission of catatonia, patients might be exposed to complications associated with unnecessarily prolonged treatment courses. A comprehensive review of the pediatric ECT literature showed an 80% efficacy rate in catatonia, which may underestimate its true efficacy in malignant catatonia as adult studies have found higher success rates in these cases [46, 47]. ECT is a remarkably safe procedure, with a mortality rate of up to 4 deaths per 100,000 treatments [48]. Since much of the morbidity and mortality associated with ECT derives from cardiac events in elderly patients, it is reasonable to suspect the procedure is even safer in younger individuals [49]. Multiple longitudinal studies in adults have found no evidence of structural, histopathological, or neurocognitive damage from ECT, even with maintenance treatments lasting several years [50–54]. In fact, use of ECT for treatment-resistant depression has been associated with increases in cortical thickness as well as increased volume of hippocampal subfields and amygdala nuclei implicated in the pathophysiology of depression [55]. More limited studies in adolescents show no differences in cognitive testing, social functioning, or school performance between ECT-treated patients and matched controls at follow-up ranging from 2 to 9 years [56, 57]. While more investigation of the long-term effects of ECT in child and adolescent patients is needed, both evidence-based clinical decision-making and a rational legal policy would balance the risks of ECT in this population against the predictable harm of foregoing a well-established and potentially lifesaving treatment of proven efficacy.

## Conclusions

We have presented the first known case of catatonia due to CsA-related neurotoxicity in a pediatric patient with SRNS. This case illustrates the importance of maintaining vigilance for signs and symptoms of CsA-related neurotoxicity (including catatonia) in patients on CsA treatment for SRNS. Moreover, it highlights the challenges faced by clinicians in jurisdictions where the use of ECT in pediatric patients is prohibited.

## Abbreviations

BFCRS: Bush-Francis Catatonia Rating Scale
BID: 2 times per day
bpm: Beats per minute
CBC: Complete blood count
CsA: Cyclosporine A
CSF: Cerebrospinal fluid
CT: Computed tomography
ECT: Electroconvulsive therapy
ED: Emergency department
FSGS: Focal segmental glomerulosclerosis
GABA: γ-aminobutyric acid
HSV: Herpes simplex virus
IV: Intravenous
MRI: Magnetic resonance imaging
NMDA: N-methyl-D-aspartate
NMS: Neuroleptic malignant syndrome
PCR: Polymerase chain reaction
PO: Oral
PRES: Posterior reversible encephalopathy syndrome
QAM: Every morning
QHS: Every night
SRNS: Steroid-resistant nephrotic syndrome
TID: 3 times per day
WBC: White blood cell
